# Convex-based lightweight feature descriptor for Augmented Reality Tracking

**DOI:** 10.1371/journal.pone.0305199

**Published:** 2024-07-18

**Authors:** Indhumathi S., Christopher Clement J.

**Affiliations:** Department of Communication Engineering, School of Electronics Engineering, Vellore Institute of Technology, Vellore, Tamilnadu, India; Purdue University, UNITED STATES

## Abstract

Feature description is a critical task in Augmented Reality Tracking. This article introduces a Convex Based Feature Descriptor (CBFD) system designed to withstand rotation, lighting, and blur variations while remaining computationally efficient. We have developed two filters capable of computing pixel intensity variations, followed by the covariance matrix of the polynomial to describe the features. The superiority of CBFD is validated through precision, recall, computation time, and feature location distance. Additionally, we provide a solution to determine the optimal block size for describing nonlinear regions, thereby enhancing resolution. The results demonstrate that CBFD achieves a average precision of 0.97 for the test image, outperforming Superpoint, Directional Intensified Tertiary Filtering (DITF), Binary Robust Independent Elementary Features (BRIEF), Binary Robust Invariant Scalable Keypoints (BRISK), Speeded Up Robust Features (SURF), and Scale Invariant Feature Transform (SIFT), which achieve scores of 0.95, 0.92, 0.72, 0.66, 0.63 and 0.50 respectively. Noteworthy is CBFD’s recall value of 0.87 representing at the maximum of a 13.6% improvement over Superpoint, DITF, BRIEF, BRISK, SURF, and SIFT. Furthermore, the matching score for the test image is 0.975. The computation time for CBFD is 2.8 ms, which is at least 6.7% lower than that of other algorithms. Finally, the plot of location feature distance illustrates that CBFD exhibits minimal distance compared to DITF and Histogram of Oriented Gradients (HOG). These results highlight the speed and robustness of CBFD across various transformations.

## Introduction

Feature detection and description are the fundamental part of many Computer Vision (CV) applications to name a few, Augmented Reality (AR) tracking [1], object detection, image retrieval, image classification, hand gesture recognition [2] and 3D reconstruction [3]. These applications process the digital image or video to obtain the feature vector from the feature descriptor. Feature vector identifies the keypoint and segregate the necessary information or pattern from the image. Infact, features reproduce the content of the image. The process of feature extraction can be divided into two steps:

- Detection which is used to find a set of keypoint from the region or patches of the image.
- Description encode the spatial localization of the keypoint for the feature computation.

Many feature descriptor algorithm have been published such as Scale Invariant Feature Transform (SIFT), Oriented Fast and Rotated BRIEF(ORB), Speeded Up Robust Features (SURF), Histogram of Oriented Gradients (HOG), Gradient Location and Orientation Histogram (GLOH), Local Binary Patterns (LBP), Fast Retinal Keypoint (FREAK), KAZE, Maximally Stable Extremal Region (MSER), and AKAZE. Each algorithm extract different features of the image such as edge, corner, blob, color and texture. These algorithm provides reliable matching and excel in specific applications like image matching, image stitching, object detection, and image classification. On the contrary, these algorithm robustness need to be improved in various conditions such as rotation, scaling, light variation, compression, blur [4] and occlusion without compromising the computation efficiency. Our model focus on the specific application named as AR tracking, so we can have a glance of the process of AR in tracking system. AR tracking has five components which is named as Input block, keypoint detector, Feature descriptor, Image matching and Pose estimator. Input block pre-process the image such as image resize, or noise removal, then detector is used to identify the necessary information of image pixel as a keypoint besides, descriptor utilize the neighbouring pixel of the keypoint to frame a feature which helps to retrieve the image. This feature is given to matching stage for the feature matching between reference and test image, once if the feature is matched, then it has to be augmented as a 3D model by the pose estimator. From this process, we can understand the significance of feature descriptor in AR tracking. The challenges of AR tracking is efficiency and robustness. Efficiency is the computation time of AR model. Robustness defines the feature extracted from the descriptor should remain same including the transformed images. Many descriptors such as handcrafted and learning based model have been published for the variety of applications in recent years. The Convex-hull based feature descriptors have been published to improve the accuracy of image classification hence, the result proves the Convex-hull [5] based model achieves good result than other models however, it observes only shape of the image and it ignores the fine details of the image which is essential to provide the more accurate results. Therefore, it lacks to observe fine information present in the image [6]. To overcome this limitation, the distributed optimization algorithm is developed. This algorithm is adopting gradient tracking and stochastic matrix confined to closed convex set in convex optimization which helps to enhance the application of privacy preservation and smart manufacturing. In fact, the distributed optimization model uses more number of variables and constraints to extract the fine details of the image which leads to utilize more computation [7]. To face this challenge, [8] author published the model, Joint Detection Tracking and Classification (JDTC), which improves the accuracy in image classification with efficient computation and it uses star convex shape to the target model which helps to identify the shape and size of the target. This convex shape implementation process with less computation and it can track the same shape with different size precisely. The learning based model such as superpoint is proposed [9] as the self supervised network model which utilize the full size image, to process the input however other models in literature uses patches of image for the feature extraction. In [10] author presented a super thermal design for the feature detection and description of thermal image. The model uses the Faster R-CNN concept to detect the features in texture less thermal images. Hence, this type of learning based model can be combined with convex model to reduce the computation. In [11] author proposed, deep Convex approximation network to generate high resolution images by utilizing residual aggregation method which improves the spatial mechanism of the model. Though the model can restore the high resolution image from low resolution, it fails to restore the edges of the input. In [12] author mentioned, the dilation projection on convex set uses iterative method to find the intersection of the convex set and reduces the error generated during the process of Computed Tomography (CT) reconstruction. This CT reconstructed image are extracted with fine edges and removes the artifacts in image. However this dilation projection faces the challenge to identify the contour with more than two convex intersection in same point. To overcome this limitation, [13] utilizes majorization-minimization algorithm which enhances the image smoothing and protect the necessary information present in the image however it lacks in computation complexity. This can be improved in [14], author adopted swarm particle optimization for image restoration as its known this bio inspired model reduces the computation. This model finds the common point of image to project the convex set. Co-variance matrix is one of the solution to the optimization problem in CV applications [15]. The author [16], designed the modified ORB descriptor for AR tracking to improve the feature description by deploying the adoptive threshold method which enriches the robustness but it limits with the computation. In [17] implemented the Unmanned Aerial Vehicle (UAV) based metaverse data collection, which adopts the convex optimization with reinforcement learning to optimize the time-sequential problem. These challenges are motivated to develop a light weight feature descriptor model. In our recent work [18] we improved the robustness of the feature descriptor for few transformations such as rotation, lighting and blur variation. In this work, our focus is on to design a lightweight feature descriptor without compromising the robustness. In this context, we proposed convex design to improve the design process of feature extraction with better accuracy in the feature description. Convex optimization is the essential tool in data analysis, modeling and statistics. However, extraction of necessary features with efficient computation is the major challenges of the above mentioned existing descriptors this can be addressed in our work. The main contribution of our work is two-fold.

1. The proposed work is implementation of Convex Based LightWeight Feature Descriptor (CBFD) for automatic prediction of features in image.
2. The quadratic polynomial predicts the feature of the image plane using co-variance matrix.

## Methodology

**Fig 1:**
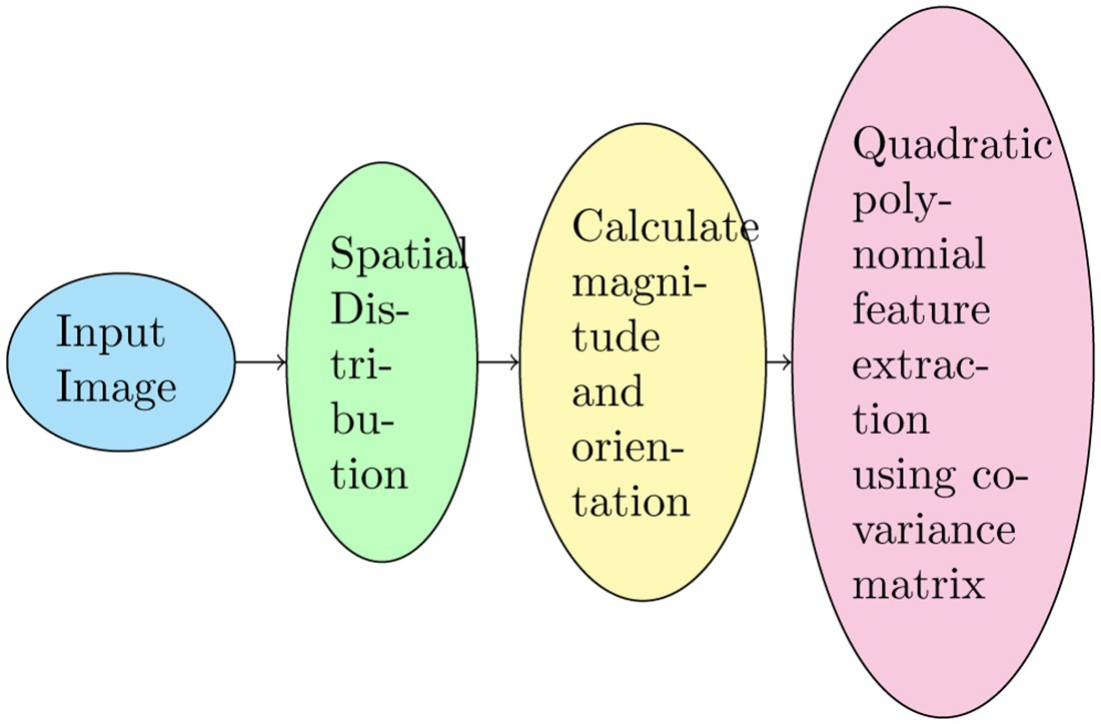
Process flow diagram of CBFD.

This section presents the detail of the proposed convex-based feature extraction model. We introduce a geometrical distribution in the 2D space to describe the image features. We propose two filters, namely, the bilateral and multilateral [18] to extract the features followed by directional orientation of the geometrical shape for feature description and the flow diagram is illustrated in Fig 1.

### Describing through quadratic polynomial

**Fig 2:**
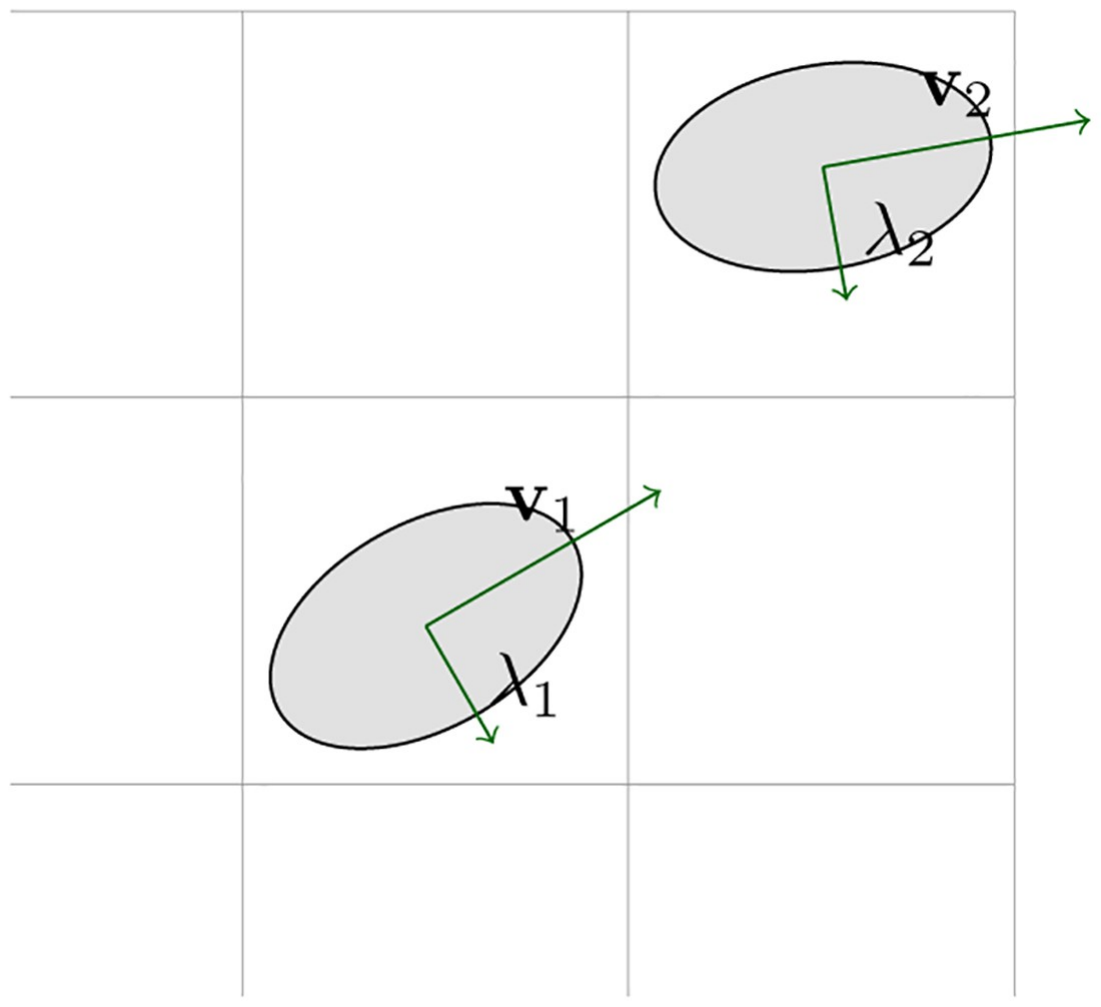
Quadratic polynomial in 2D image plane.

**Fig 3:**
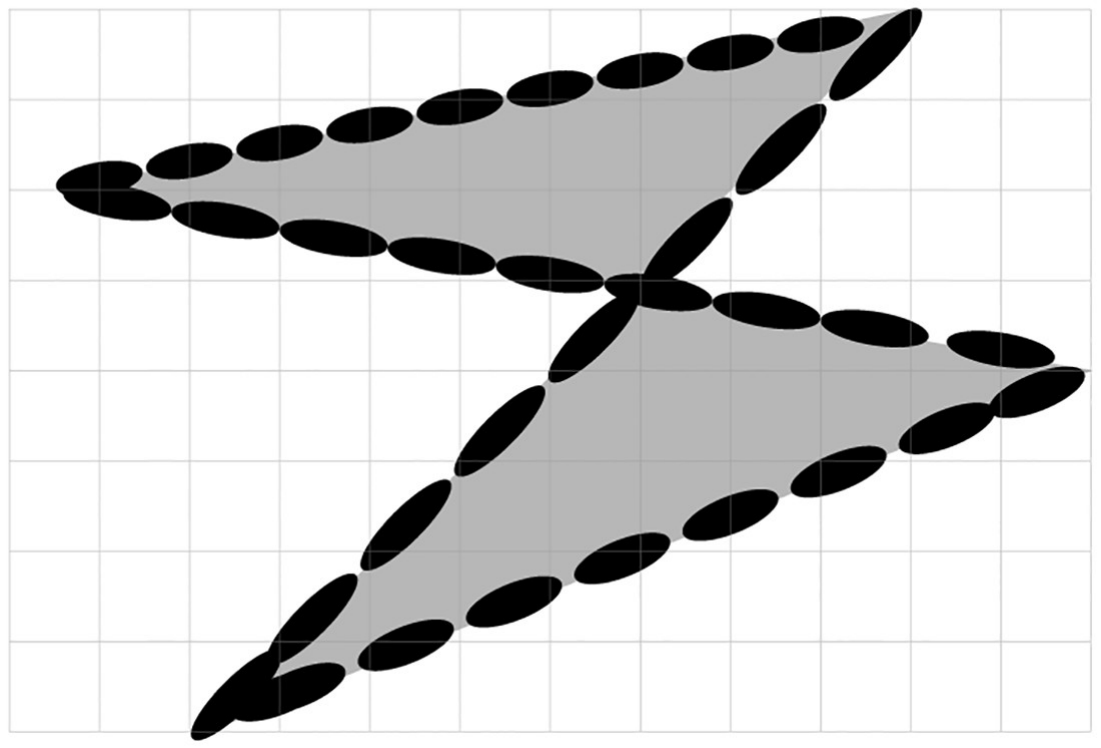
Sample image for illustration.

As the details present in image are represented by the variations in pixel intensities, it should be possible to exhibit these variations in terms of polynomial equation, provided its coefficients are found proportional to the intensity variations. There are various polynomials exist, namely constant, affine, quadratic and cubic. Of these, affine and quadratic are of degree 2, and are less in complexity, while others are of degree more than 2. As Image is a 2D plane, it could be less complex and it is suffice to choose affine or quadratic to describe the image details. Considering the significance of degree of freedom, we have chosen, a quadratic polynomial than affine for feature description. The chosen quadratic polynomial is

$$\begin{array}{c} x^{T}Rx-2x_{c}^{T}Rx+x_{c}^{T}Rx \end{array}$$ (1)

where $x\in R^{2}$, $x_{c}\in R^{2}$ and $R\in R^{2\times 2}$. the above equation can also be written as

$$\begin{array}{c} {\left(x-x_{c}\right)}^{T}R\left(x-x_{c}\right) \end{array}$$ (2)

represents a quadratic form, where $R=[\begin{array}{cc} \sigma_{x}^{2} & 0\\ 0 & \sigma_{y}^{2} \end{array}]$ is a symmetric and positive-definite matrix, $\sigma_{x}^{2}=K$ and $\sigma_{y}^{2}=∊$, *K* indicates block size, *ϵ* > 0 is small number. The axes length laid out in 2D plane (2) can be determined from the eigenvalues and eigenvectors of the matrix **R**. Specifically, the eigenvectors give the directions of the axes, and the square roots of the corresponding eigenvalues give the lengths of the semi-axes. Let **R** have eigenvalues λ_1_ and λ_2_, and let **v**_1_ and **v**_2_ be the corresponding normalized eigenvectors. Then, the axes length in either direction is given by $a=\sqrt{\lambda_{1}}$ with direction **v**_1_ and $b=\sqrt{\lambda_{2}}$ with direction **v**_2_ respectively. The image plane is illustrated in Fig 2. While these axes represent the lengths of the two principal axes of the geometry, it is to be noted that the actual axes lengths in the coordinate system are obtained by transforming these lengths back to the original coordinate system using the eigenvectors. Fig 3 illustrated the process of extracting the features from highlighted shape. In Fig 3, grids denote the size of sub blocks [19] used for processing. We also observe that the resolvable features are identified only at some spatial locations in 2D plane. It is observed from Fig 3 that the grids of interest is spanned by a polynomial given in Eq (2). In Fig 3, the polynomial structure is aligned in the grid of interest so as to track the revolvable regions of interest. It depends on the nature of the image. In summary, it is required to

1. Distinguish the resolvable regions of interest. For this purpose, we develop an algorithm that exploit the pixel intensity variations.
2. Figure out the algorithm that governs the alignment of polynomial shown in Fig 3 in grids of interest.

The above steps demand an optimization method, with appropriate objective function having well established constraints. So, the question is, how to establish the optimization method which is controlled by the identified regions of interest and pixel intensity variations. We discuss them in detail in the next section.

### The optimization problem

This section describes the optimization of feature extraction using polynomial function. The **v**_1_ and **v**_2_ are the eigen vectors of the **R** matrix shown in (2). $\sqrt{\lambda_{1}}$ and $\sqrt{\lambda_{2}}$ decides the span in the sub blocks. It is important to make the span compact within the sub blocks. It is nothing but the trace of **R**, as its diagonal elements are proportional to the eigen values that decide the axis length. It also decides the overlapping of the polynomials in the neighbouring sub blocks, which avoids repetition, if any. So, it is needed to minimize the trace of **R**. Moreover, one of the constraints is that **R** must be positive definite as highlighted in section 1. So far, the objective function with the constraint is identified, however, the constraint on **R** that decides the orientation is yet to be found, so that the alignment can be automated according to the pixel intensity variation. The direction of the features can be identified from the variation in pixel intensity. We propose two filters, namely, Bilateral and Multilateral that take care of obtaining the intensity variation and orientation, the former one is denoted as **f**_**y**_ and the latter is **f**_**x**_. To illustrate how these filter operate on an image matrix, we consider the gray scale sample image matrix represented as **I**

$$\begin{array}{c} I=[\begin{array}{cccc} i_{11} & i_{12} & \cdots & i_{1N}\\ i_{21} & i_{22} & \cdots & i_{2N}\\ \vdots & \vdots & \vdots & \vdots \\ i_{M1} & i_{M2} & \cdots & i_{MN} \end{array}] \end{array}$$ (3)

For the given filter of size *k* × *l*, there can be a vector defined out of (3) as follows

$$\begin{array}{c} i=[\begin{array}{cccccccccc} i_{11} & \cdots & i_{1l} & i_{21} & \cdots & i_{2l}\cdots & \cdots & i_{k1} & \cdots & i_{kl} \end{array}] \end{array}$$ (4)

Now, we would like to define the metrics *G*_*pq*_ and *H*_*pq*_, needed to describe the intensity variations, for the varying $p=1,2,\ldots ,M-(\frac{k}{2}+1)$ and *q* = 1, 2, ⋯, *N*−(*l* + 1), in addition, when *s* = 1, 2, ⋯, *k* and *t* = 1, 2, ⋯, *l* we define the vectors,

$$\begin{array}{c} f_{y}=\{\begin{array}{ccc} 1 & ; & s=1\;|\;t=2,3\cdots ,l-1\\ -1 & ; & s=k\;|\;t=2,3\cdots ,l-1\\ 0 & ; & \text{otherwise} \end{array} \end{array}$$ (5)

$$\begin{array}{c} f_{x}=\{\begin{array}{ccc} 1 & ; & s=1\;|\;t=2,3\cdots ,k-1\\ -1 & ; & s=l\;|\;t=2,3\cdots ,k-1\\ 0 & ; & \text{otherwise} \end{array} \end{array}$$ (6)

Then

$$\begin{array}{c} G_{pq}=\sqrt{{\left(i\cdot f_{x}\right)}^{2}+{\left(i\cdot f_{y}\right)}^{2}} \end{array}$$ (7)

$$\begin{array}{c} H_{pq}=\text{arctan}(\frac{i\cdot f_{y}}{i\cdot f_{x}}) \end{array}$$ (8)

It is needed that *G*_*pq*_, *H*_*pq*_ impact the optimization problem under discussion. The **R** matrix mentioned in (1) satisfies the requirement only in the first sub block, yet it is not sufficient, when the operation window is moving further. The matrix **R** will undergo rotation and spanning to meet the further requirement discussed earlier. This principle lay a foundation to formulate a objective and constraint in the optimization problem. The diagonal matrix derived from **R** should also be positive definite and must be processed through $Z=[\begin{array}{cc} \text{cos}(H_{pq}) & -\text{sin}(H_{pq})\\ \text{sin}(H_{pq}) & \text{cos}(H_{pq}) \end{array}]$ to give **Q** with positive definiteness and provides the degree of polynomial to satisfy the all requirements. By doing so, the polynomial axis length are still the Eigen values of **R**, while the directions are oriented by transforming the original Eigen vectors. Putting together, the optimization problem is formulated as

minimize:trace(Q)subject to:Q=QT≻0 (9)

From these constraints, the alignment of the polynomial is automated in our model with respect to the change in pixel intensity of the image. The working principle of CBFD is shown in Algorithm:1.

**Algorithm 1** An algorithm for Convex Based Lightweight Feature Descriptor

**Require: I**_*m*×*n*_, *K*, *ϵ*, **R**

**Ensure:** $\sigma_{x}^{2}\leftarrow K,\sigma_{y}^{2}\leftarrow ∊$

 Output **W** = **0**_*M*×*N*_

 **for** *p* = 1 to $M-(\frac{k}{2}+1)$ **do**

  **for** *q* = 1 to *N*−(*l* + 1) **do**

   Obtain *f*_*x*_ from (6)

   Obtain *f*_*y*_ from (5)

   Calculate *G_pq_* as in (7)

   Calculate *H_pq_* as in (8)

   Calculate *Z* using *G*_*pq*_ and *H*_*pq*_

   Execute the optimization of (9) to get **Q**.

   Span the polynomial in **W**_*pq*_ with appropriate orientation using **Q**.

  **end for**

**end for**

## Results and discussion

**Fig 4:**
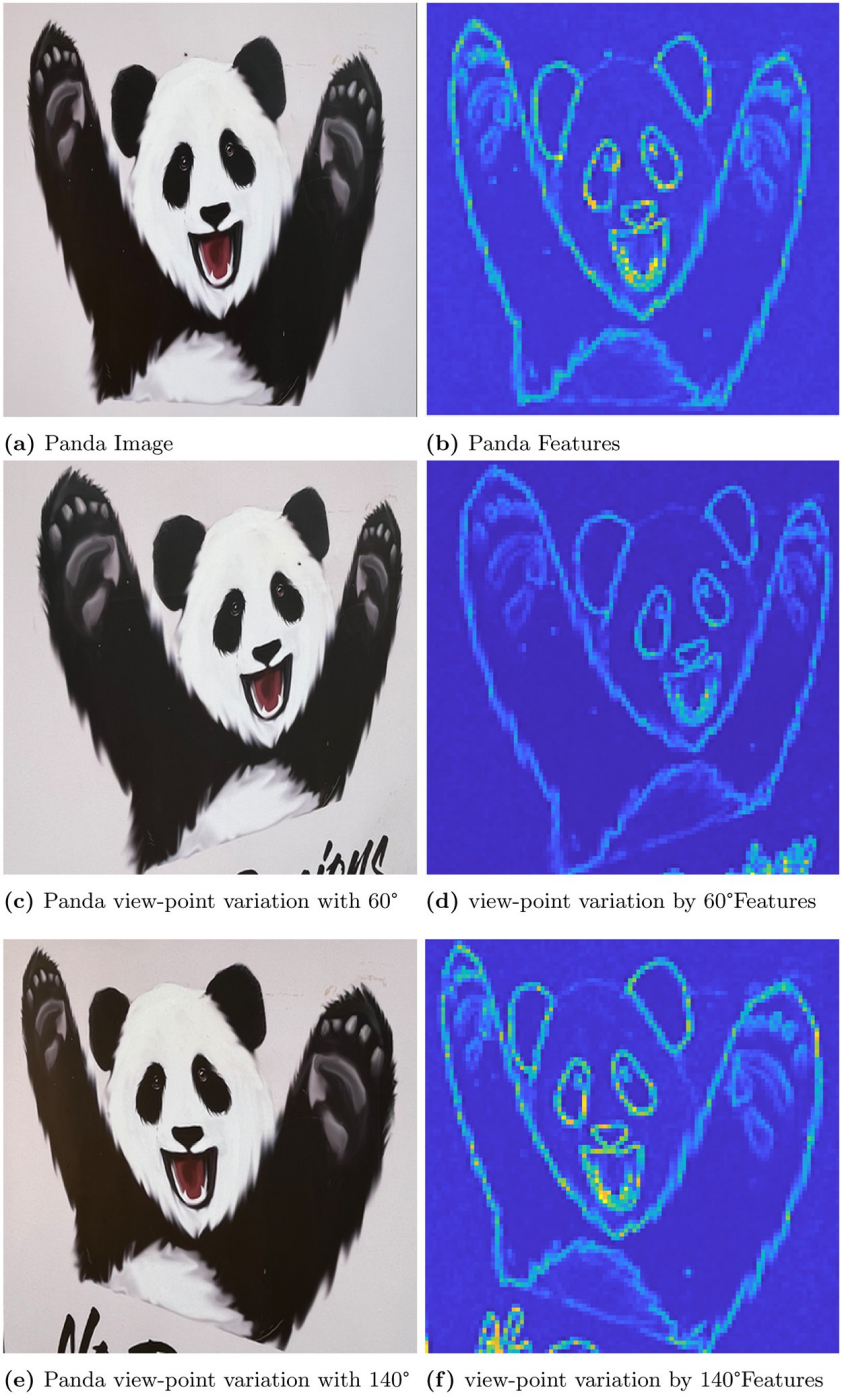
Illustrating the effectiveness of CBFD considering the original image and its view point variation. (a) Panda Image, (b) Panda Features, (c) Panda view-point variation with 60°, (d) view-point variation by 60° Features, (e) Panda view-point variation with 140°, (f) view-point variation by 140° Features.

**Fig 5:**
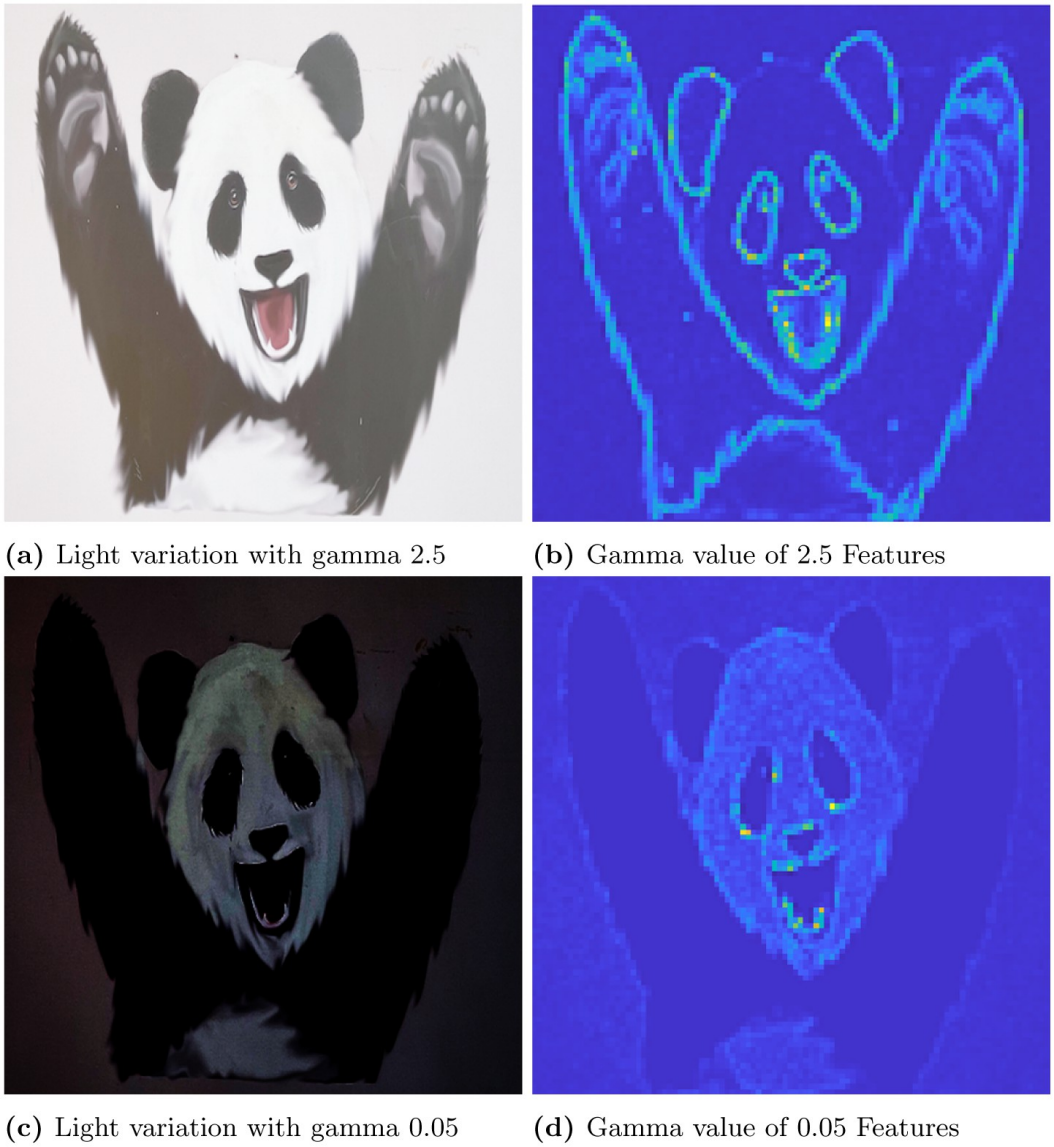
Illustrating the effectiveness of CBFD considering the original image and its light variation. (a) Light variation with gamma 2.5, (b) Gamma value of 2.5 Features, (c) Light variation with gamma 0.05, (d) Gamma value of 0.05 Features.

**Fig 6:**
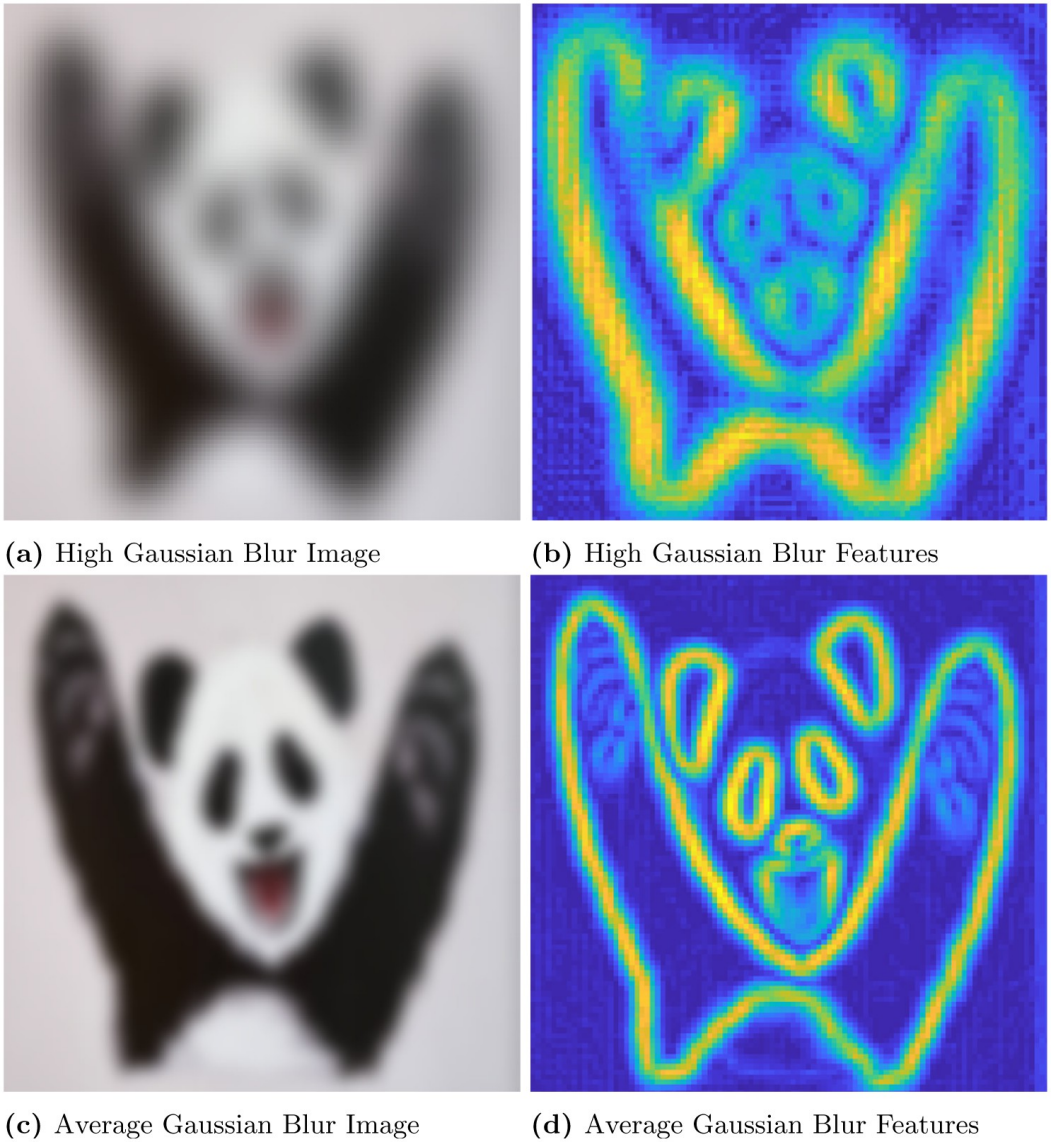
Illustrating the effectiveness of CBFD considering the original image and its blur variation. (a) High Gaussian Blur Image, (b) High Gaussian Blur Features, (c) Average Gaussian Blur Image, (d) Average Gaussian Blur Features.

**Fig 7:**
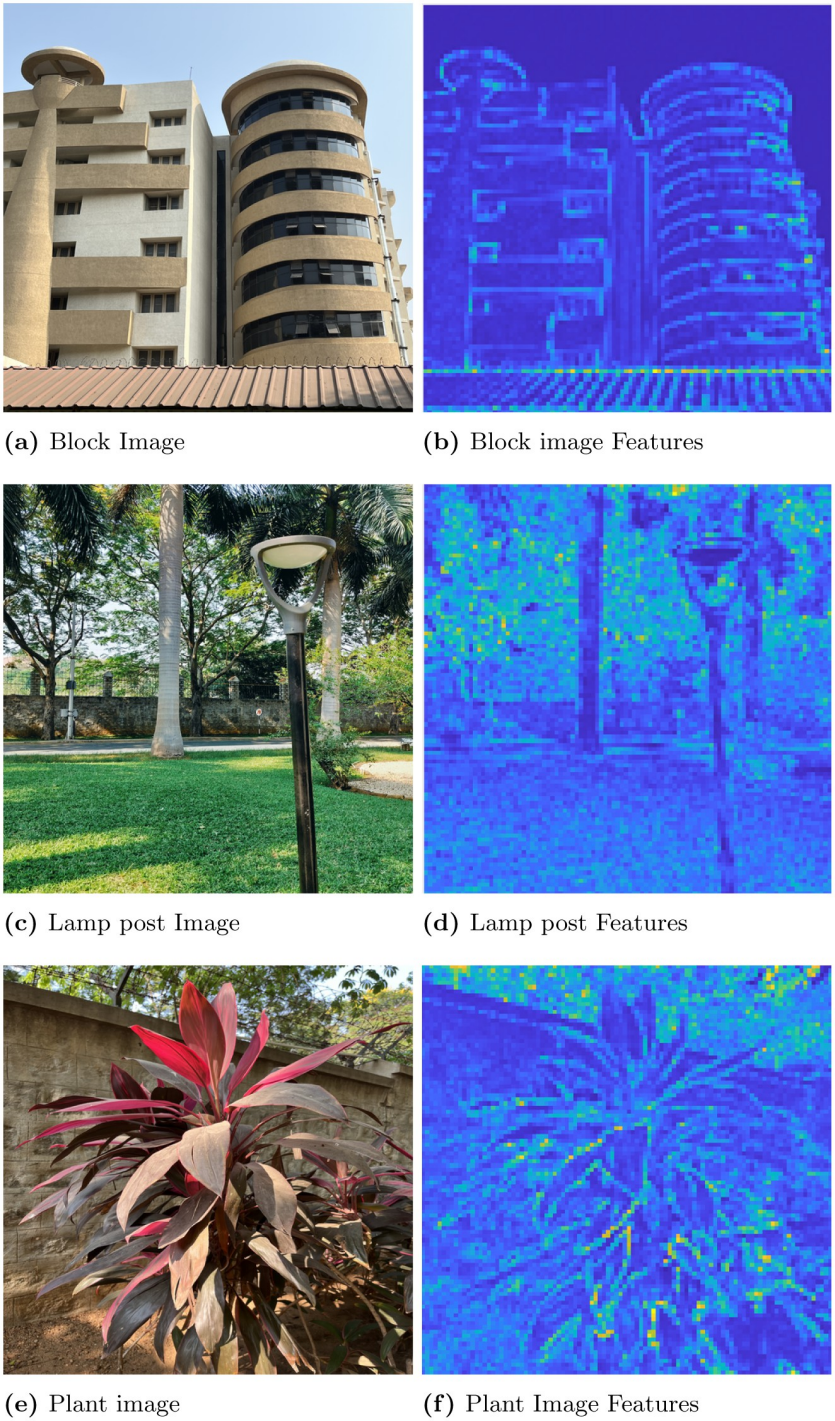
Feature extraction of complex scenes. (a) Block Image, (b) Block image Features, (c) Lamp post Image, (d) Lamp post Features, (e) Plant image, (f) Plant Image Features.

**Fig 8:**
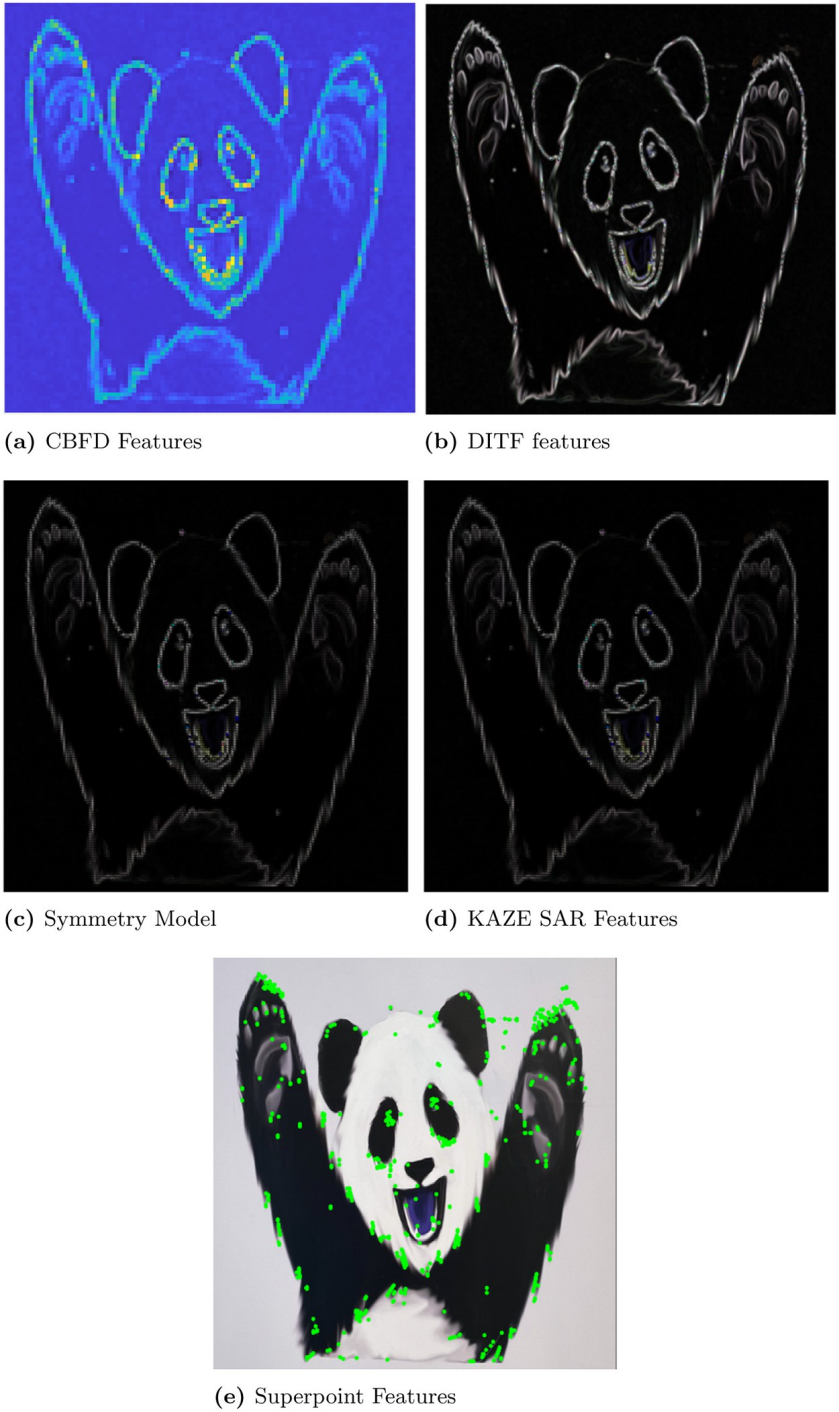
Comparison of convex and non-convex optimization. (a) CBFD Features, (b) DITF features, (c) Symmetry Model, (d) KAZE SAR Features, (e) Superpoint Features.

In this section, first we discuss the results of convex optimization based feature extraction, followed by the comparison analysis of existing feature descriptors. We experimented with four different images to analyse the robustness of the algorithm. In that case, the three images are block, lamp post and plant image. The block image has many glass windows with more number of edges. A Lamp post is situated in garden with plenty of trees and walls in a background of the image. The plant image with red leaf has bunch of leaves with abundance of edges includes the wall background. These images are used to test the stability of the feature extraction by CBFD in complex scenes. Another image is ‘panda’ which has plain background so it is used to test the transformation of image such as view-point variation, light and blur variation. The ‘panda’ view-point is varied with the angle of 60°and 140°. In the account of light variation gamma correction is adopted to the image, this provides the brightness and darkness variation to the image as per the gamma value. Hence, the *γ* = 2.5 which enhances the brightness of the image and when *γ* = 0.05 it creates the darkness to the image. The blur variation creates the image with less visibility. This blur in image is also called as a noise which is formed due to the gaussian filter, this filter adopts the gaussian function to distort the image quality with different level of noise such as noise value of *σ* is 5 and 2 which reflects in the image given in Fig 6. At this instance, it is difficult for the model to extract the features from transformed images so we have included these transformation to enrich the CBFD model. All the images are used in CBFD is in the uniform size of 512 × 512 respectively. The grid size of the input image is fixed as K = 3 to validate the accuracy. So that the grid is not get overlap with the neighbouring pixels. The pixel intensity variation of the grid is obtained from the Eqs (7) and (8). The span of the grid of interest have the impact on **R**. The diagonal values of the co-variance matrix governing the degree of polynomial in the grid of interest is chosen as $\sigma_{x}^{2}=9,\sigma_{y}^{2}=0.04$. Panda image and its output feature is shown in Fig 4a and 4b. From the result, we can observe the plot is described in color map is for better visualization of the pixel intensity variation. The changes in pixel intensity has different extent, each values are projected with its corresponding color which indicates the significant features of the image. The color map is chosen in such a way that the blue shaded one is of lowest significance in terms of feature intensity and yellow is of high significance. In Fig 4a, the color changes from white to black and edge of the image has drastic variation in intensity so it reflected as yellow color in output feature of Fig 4b, which indicates the mouth, eye corner, and curve portion of body, then the remaining extracted portion of panda are in low intensity variation hence the features are in blue indication. Our convex method automatically predicts the degree of polynomial for every grid as mentioned in section 2. The intensity may be low or high but our model predicts all the features, which reflects the shape of the panda image. The validation of rotation transformation, is the significant property of AR tracking and its illustrated in Fig 4d and 4f. The panda image tested with view-point variation of 60 and 140 respectively. The rotational image feature has high pixel intensity variation than the original image of panda so it is highlighted with more remarkable features with yellow color. Even though image is rotated if we compare the features of Fig 4b, 4d and 4f the features predicted by the model remains same. This shows the robustness of rotation transformation by the CBFD model. Fig 5 shows the light variation of image with respect to gamma value. Gamma adjust the brightness level of the image, higher the gamma value enhances the brightness and low value leads to dim the light. Thus, the *γ* = 2.5 indicates the brightness of the image which is shown in Fig 5a and its feature extraction are illustrated in Fig 5b it shows CBFD retains the shape of the image in light variation. However, when the *γ* = 0.05 is attained its difficult for human eye to perceive the vision but the model managed to retrieve the mouth, nose and eye of the panda image. In a similar way, the blur variation of the image is illustrated in Fig 6 is examined by CBFD. As mentioned earlier in this section gaussian filter is used to vary the noise level which leads to induce the blur variation in image when the *σ* is 5 it indicates the high blur value to the image which is illustrated in Fig 6a as expected CBFD predicts all the features to retrieve the shape of the image. Fig 6b shows the features in yellow color due to high pixel intensity variation in image the similar way CBFD extract the features of average noise level such as *σ* = 2 present in Fig 6d. Therefore, the results of affine transformations proves the CBFD regains all the features in view-point variation, light and blur variation. For further analysis of complex scenes, we have included three images which is taken in three different scenes. The feature extraction of block, lamp post and plant images are shown in Fig 7. Even if we change the input image, our model predicts the robust feature irrespective of change in the nature of the image. In Fig 7d, While looking at the lamp post image, our human eye perceives the significance of intensity variation in the regions, namely, grass, trees and edges of the wall. The presented features in the results of lamp post image also convey the same message. From the color map information, these areas are shaded with the yellow color to show the significance. Other background information in the original lamp post image is monochromatic color, which is of least significance, when human eye perceives it. They are highlighted in the feature description with the help color mapping in blue color, which is of low significance. The Fig 7b block image consist of more intensity variation with the glass window and roller shape of the image which highlights the high significant feature as in yellow and the flat surface in the block and background sky which consist of low intensity and reflected with blue color in output. In Fig 7f illustrate the plant image feature extraction, the yellow color indicates the fine features present in the image as a edge of the leaf and wall roof followed by the coarse information of leaf and background are reflected with blue variation. From this, we can conclude our model predicts the intensity variation precisely. This intensity variation retrieves the edge of the image. The validation of proposed model is compared with existing model is illustrated in Fig 8. In [9] author trained the self supervised network model to automatically identify the features and they have obtained 0.98 as mean average precision considering 10 categories of images. However, we have considered the three types of images and obtained a average precision of 0.97, which is compared with the precision of proposed algorithm. The DITF model [18] extract the features using block normalization and orientation of block is measured by dominant gradient of the feature. In [20] the authors have implemented the phase symmetry model to identify the hyperbola and they used HOG descriptor to measure the features present in the image. Modified SURF with KAZE descriptors [21] have been published to recognize the edge feature using second order differential equation without affected by the noise. From the Fig 8 we can spot the intensity variation is clearly identified from our proposed model features which is shown in Fig 8a. The proposed model results are generated from polynomial orientation which is not implemented in the existing method.

**Fig 9:**
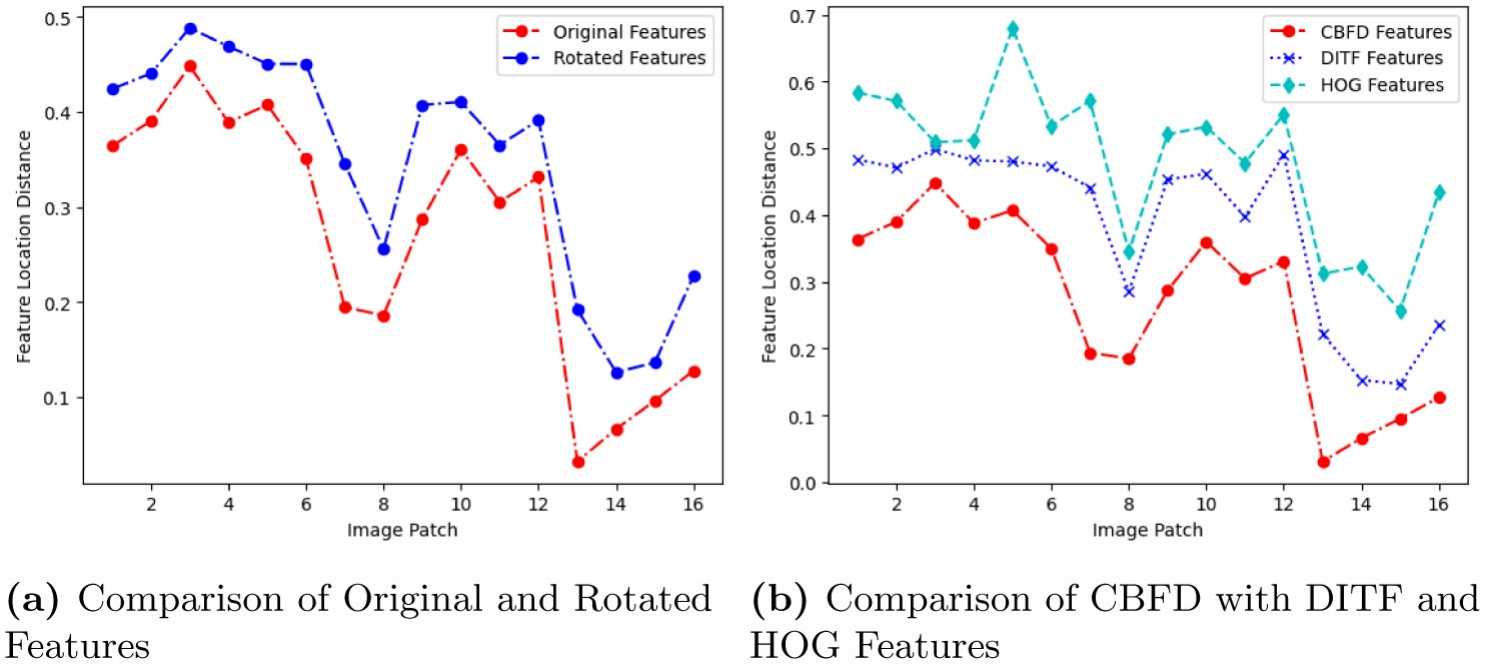
Comparison of feature location distance for different descriptor. (**a**) Comparison of Original and Rotated Features, (**b**) Comparison of CBFD with DITF and HOG Features.

The analysis of feature extraction is extended to feature localization in image. This localization is observed from the feature location distance between two images. This measurement is experimented using the panda image. The image is divided into *A* patches, with *A* = *A*_1_ × *A*_2_, *A*_1_ vertically and *A*_2_ horizontally. For the simulation, we have chosen *A*_1_ = 4 and *A*_2_ = 4 so four patches are in vertical plane and four patches are in horizontal plane then CBFD algorithm is applied to every patch to find the feature location. The feature location of each patch is compared with the euclidean distance between reference and test images. Consequently, when the distance between reference and test image is closest we can conclude the feature location is almost same or matches with each other at instance that distance has to be 0. However, in practical case, there will be a deviation in the location distances so the value will be in positive and non-zero. Similarly, the rotation in-variant features are identified and located in the image, this result is illustrated in Fig 9a. From the Fig 9a the distance of original feature and rotation feature is very small which indicates CBFD is robust in rotation transformation for the feature prediction. At the end, we compared CBFD with DITF, and HOG feature localization, from the results in Fig 9b, CBFD curve is not increases beyond the value of 0.5 while the DITF and HOG score achieves the range of 0.7 in terms of location distance. Hence, it proves CBFD model surpasses the DITF and HOG features in feature extraction.

**Fig 10:**
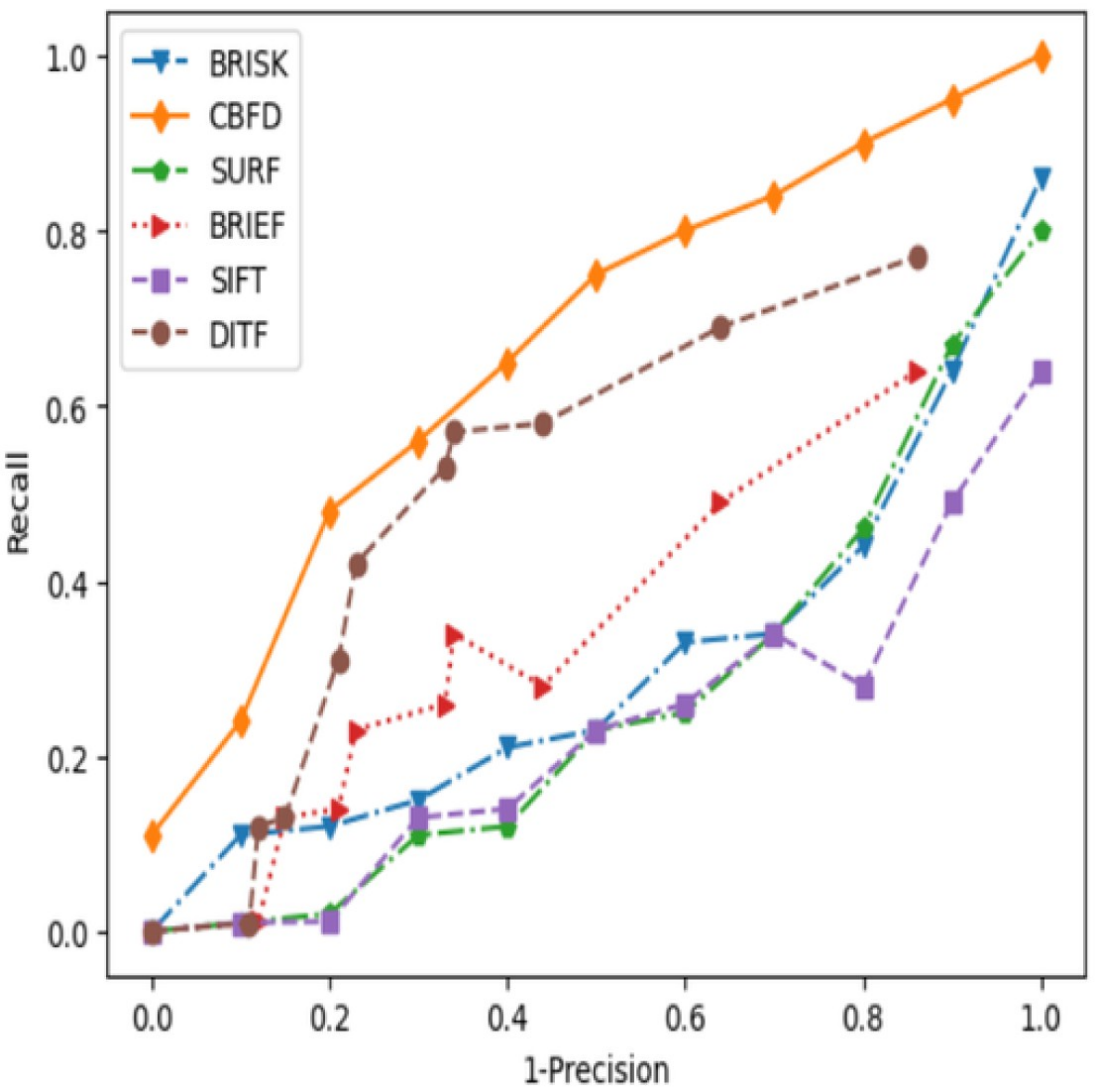
Precision recall curve.

In Machine Learning model, the performance of the feature extraction is measured using precision and recall. This is considered as a standard metric so we experimented both precision and recall for three different types of images such as panda image, Lamp post and plant image respectively, which helps to identify the image retrieval of the model. The average precision and recall of several models are shown in Table 1. The CBFD achieves high average precision value of 0.97 for the test image, compared to SUPERPOINT, DITF, BRIEF, BRISK, SURF and SIFT which obtain the scores of 0.95, 0.92, 0.72, 0.66, 0.63 and 0.50 respectively. In a similar fashion CBFD’s recall value of 0.87 representing at the maximum of a 13.6% improvement over existing descriptors. The precision and recall value of proposed model is surpasses the existing model for all three types of images. Since, the nature of the images is different for three images CBFD model generates almost similar precision and recall value than the existing descriptors. This result proves the robustness of the CBFD model in feature extraction. Fig 10 shows the estimated precision recall curve of different descriptors for the panda image. For ideal case, the curve will rise and will saturate at the maximum value of 1. However, in real time applications due to noise and the image quality its difficult to attain ideal curve. From the illustration of our curve, CBFD model attains the good retrieval compare to the existing models. In addition to that, to strengthen the performance evaluation of CBFD model matching score is also included. The matching score is used to find the similarity between the features of two images. One of the images is a reference image, which has not undergone any transformation. The features of the original and rotated image are extracted by CBFD and the correspondence of the two images are compared for an image matching. The comparison result provides the similarity between two images. The number of such a matching feature out of total features indicates a score, meaning that if all features of original image are matched with the features of rotated then the matching score is 1 and if there is no similarity the score is 0. For the validation of the CBFD model the four types of image features are compared with their rotated images with the angle of rotation being 60. The similarity or correspondence between these two images are shown in Table 2 is 0.98, 0.97, 0.97 and 0.98 for the panda image, lamp post, block and plant images respectively This outcome proves CBFD model notably achieves best results in feature extraction. During the optimization of first constraint the model escalated the new constraint to improve the model which will be discussed in next section.

### Improvement constraining the requirement on lateral filter sizes

**Fig 11:**
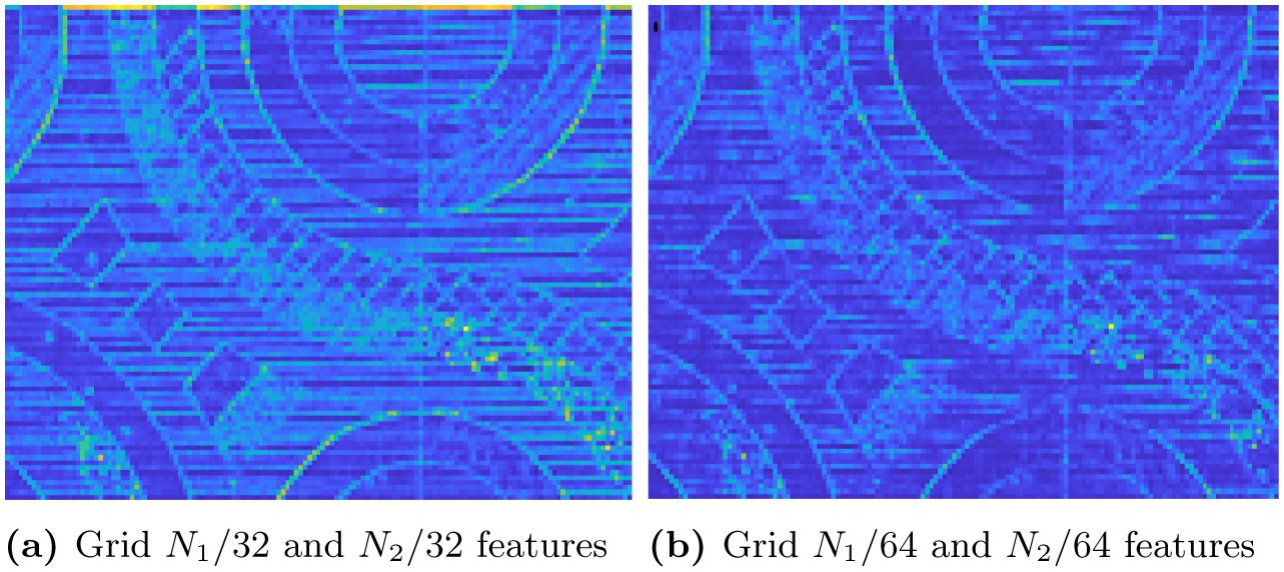
Grid size optimization. (a) Grid *N*_1_ = 32 and *N*_2_ = 32 features, (b) Grid *N*_1_ = 64 and *N*_2_ = 64 features.

It is known that human eye can perceives the nonlinear portion of an image in terms of infinitesimal linear sections. As long as the resolution is not affected, the combinations of linear sections can yet represent the nonlinear portions. The number of pixels used to change this resolution is found as *N*_1_/64 and *N*_2_/64 [22] where the *N*_1_ and *N*_2_ are the size of the source image and *Λ* is represented as $\text{diag}(\frac{N_{1}}{\lambda },\frac{N_{2}}{\lambda })⊙R$. The ⊙ represents a dot product between the elements. In the previous optimization problem, that is from first constraint, we have not considered the effect of this approximation. Instead, we have fixed a grid size of some static value. It is to be noted that the grid size changes the direction and length of the polynomial, which in turn is the covariance matrix. The optimization problem is constructed as in 10. Fig 11a shows the feature extraction of the grid size (*N*_1_/32,*N*_2_/32) and Fig 11b presents the features for the grid size of (*N*_1_/64,*N*_2_/64) from the visualization it is clear that (*N*_1_/64,*N*_2_/64) features are failed to retrieve all the features. However, shape of the image can be retrieved in (*N*_1_/32,*N*_2_/32) grid. So, maintaining the grid size beyond the level of (*N*_1_/64,*N*_2_/64) is not advisable. So, this can be considered as the additional constraints into the optimization problem to design a lightweight feature descriptor.

**Table 1:** The average precision and average recall—A comparison of proposed algorithm with other algorithms on three different images.

Descriptor	Panda image		Lamp post image		Plant image	
	Average Precision	Average Recall	Average Precision	Average Recall	Average Precision	Average Recall
**CBFD**	**0.99**	**0.91**	**0.98**	**0.90**	**0.96**	**0.82**
SUPERPOINT	0.97	0.78	0.95	0.73	0.94	0.71
DITF	0.94	0.76	0.92	0.73	0.90	0.70
BRIEF	0.78	0.72	0.70	0.67	0.68	0.65
BRISK	0.69	0.67	0.66	0.64	0.64	0.52
SURF	0.68	0.66	0.63	0.52	0.60	0.46
SIFT	0.59	0.56	0.50	0.46	0.43	0.41

**Table 2:** A matching score of CBFD with four different images.

Test Image	Matching score
Panda Image	0.98
Lamp post image	0.97
Block image	0.97
Plant image	0.98

The computation time is an essential factor for AR tracking. To overlay the 3D model in real world AR tracking system should be faster. The tracking time should not to be more than 100ms for the effective tracking system. Despite of many levels in the process as mentioned in section:Introduction. our focus is the development of the descriptor with good computation. Therefore, Table 3 shows the comparison analysis of computation time for CBFD with existing descriptor. The computation time of the CBFD is calculated from *M*—(*K*/2 + 1) + *N*-(*l* + 1) ⋅ $\sqrt{C}$ ⋅ $O{\left(n\right)}^{3}$. The image size *M* = 640, *N* = 800 and *K* = *l* = 3 then *n* is 2, it represents the number of variables then *C* indicates the number of linear inequalities which is 2 in our model. Hence, from this evaluation, we obtain the computation time of CBFD as 2.8ms it is faster than other algorithms. The second high value holds by DITF descriptor then third position is for BRIEF but it does not have its own detector for feature detection so changes in the detector leads to fluctuate the computation time and it also lacks in affine transformation property. The CBFT algorithm describes features by sliding a window of small patch all across the entire image. To solve this limitation, we will try to incorporate finding the descriptor of an entire image all at once. As far as the practical implementation of CBFD is considered, it can be well operated in NVIDIA Jetson Orin Nano 8GB development kit. It is a low power consuming board to implement a high level of machine learning applications. It has the capacity to run 40 Trillion operation per second. As the CBFD model is less complex and running in milliseconds time, it is very much possible to implemented CBFD in “Nano” board.

minimize:trace(λ)subjectto:λ≻0λ=λT (10)

**Table 3:** Comparison analysis of computation time.

Feature Descriptor	Computation Time(ms)
**CBFD**	**2.8**
DITF [18]	3.0
BRIEF [23]	3.8
BRISK [24]	10.6
SURF [23]	111.7
SIFT [25]	448.6

## Conclusion

We introduced a new approach for feature extraction using automated polynomial orientation. This approach uses convex based geometry for each spatial grid of interest to extract the features. The magnitude and orientation of the grid location is measured by the bilateral and multilateral filters. The orientation of the feature is controlled by co-variance matrix. The CBFD model addressed the optimization of feature extraction in terms of computation time which is essential for Augmented Reality Tracking. We have also shown the optimal block size which can retain the resolution of an image, when the image undergoes convex polynomial. The results have validated its usefulness. The CBFD model is verified by precision, recall and matching score. Additionally, CBFD validated the localization of the feature in image using feature location distance between two images. Compare to existing method CBFD generates robust feature extraction with less computation time including rotation, light and blur in-variance property. This work may provide the guidance for the optimized feature descriptor model. The extension of our work can address the feature extraction of scale variation and occlusion prediction.
